# Ophthalmologic identification of cerebral malaria in adults

**DOI:** 10.3205/oc000035

**Published:** 2015-11-02

**Authors:** Catarina Areias Pedrosa, Cristina Santos, Inês Coutinho, Maria Lisboa, Susana Teixeira, Filomena Silva, Graça Pires, Isabel Prieto

**Affiliations:** 1 Ophthalmology Department, Hospital Prof. Doutor Fernando Fonseca E.P.E., Amadora, Portugal

**Keywords:** malarial retinopathy, malaria, encephalopathy

## Abstract

**Objective:** To report the clinical presentation of malarial retinopathy in an adult, emphasizing the importance of this diagnosis for the clinical suspicion and prognosis of cerebral malaria.

**Methods:** A 39-year-old caucasian man presented with hemolytic anemia, thrombocytopenia, acidemia and acute renal failure, developing severe encephalopathy. The diagnosis of *Plasmodium falciparum* malaria was done and after systemic stabilization, the patient noticed a central scotoma in the left eye. Ophthalmological examination revealed retinal features of malarial retinopathy.

**Results:** At one-month follow-up, the patient had improved his systemic condition and the left eye scotoma had disappeared. Visual acuity was 20/20 in both eyes and on examination almost all lesions had regressed.

**Conclusion:** Malarial retinopathy is a diagnostic factor and a prognosis indicator of severe *P. falciparum* infection, usually with brain involvement. The knowledge of the ophthalmological features associated with severe malaria, which is more frequent in children but can also occur in adults, becomes imperative in order to reduce the risk of neurologic sequelae and associated mortality.

## Introduction

Encephalopathy is a malaria poor prognostic factor, associated with a high mortality rate [1]. In endemic areas, the diagnosis of cerebral malaria is hampered by the high rate of *Plasmodium falciparum* parasitemia not associated with disease, commonly leading to underdiagnosis [2].

In cases with brain involvement, the ophthalmological manifestations associated with malaria diffuse encephalopathy are an important diagnostic feature as it can confirm the diagnosis of this condition allowing the reduction of the risk of associated neurologic sequelae and mortality [2], [3]. We present a case of malaria encephalopathy with characteristic ophthalmological features which highlights the importance of this diagnosis for the clinical suspicion and prognosis of cerebral malaria, in order to provide an early and targeted treatment for this condition. 

## Case description

**Figure 1:**
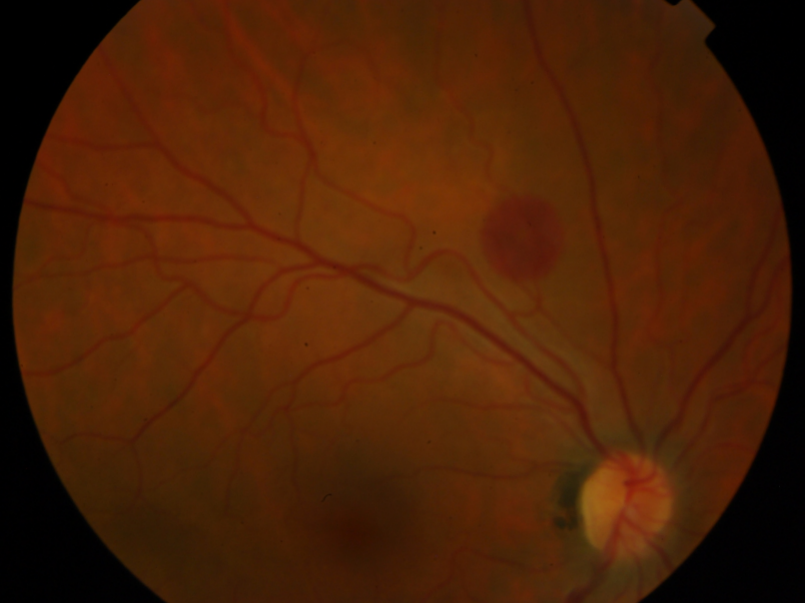
Color fundus photograph of the right eye showing a round deep layer retinal hemorrhage in the superior temporal arcade

**Figure 2:**
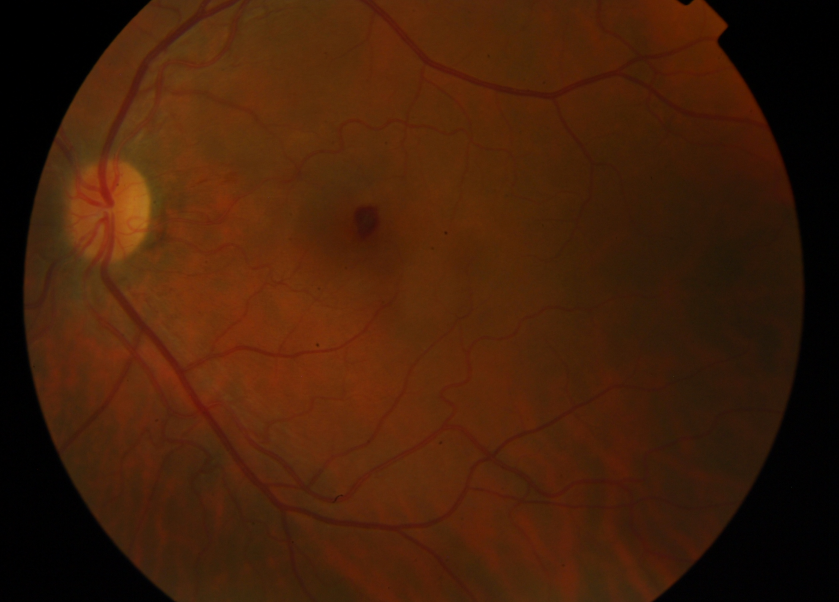
Color fundus photograph of the left eye showing foveal hemorrhage and several superficial hemorrhages in the interpapillomacular bundle

**Figure 3:**
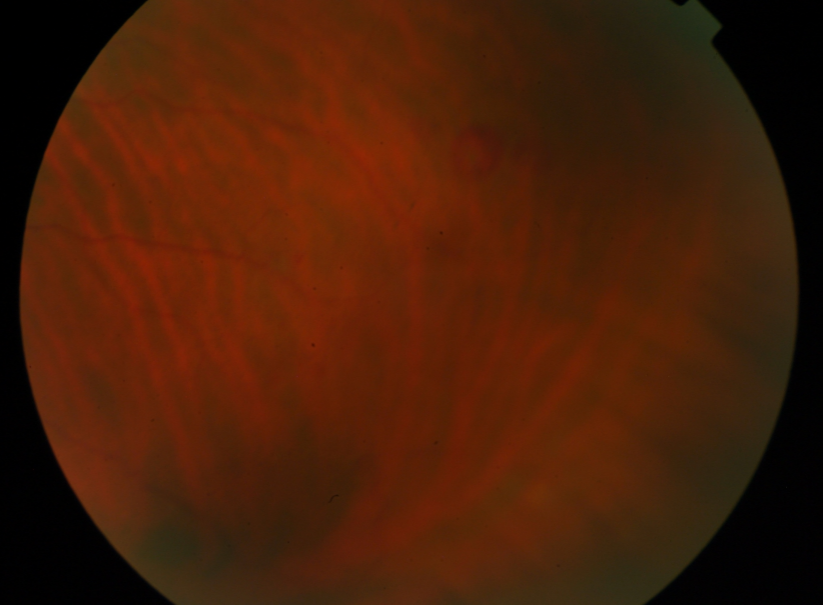
Color fundus photograph of the left eye showing a deep white-centered peripheral hemorrhagic lesion

**Figure 4:**
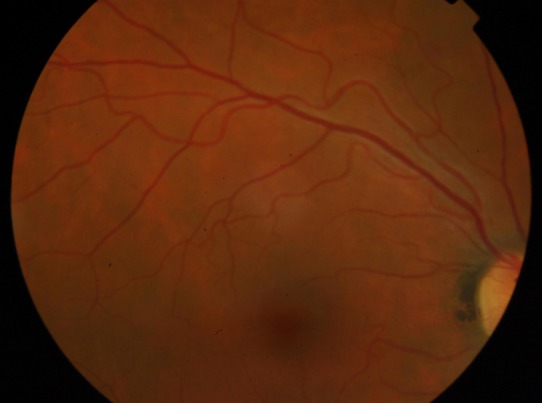
Color fundus photograph of the right eye, at one-month follow up, showing regression of retinal lesions

**Figure 5:**
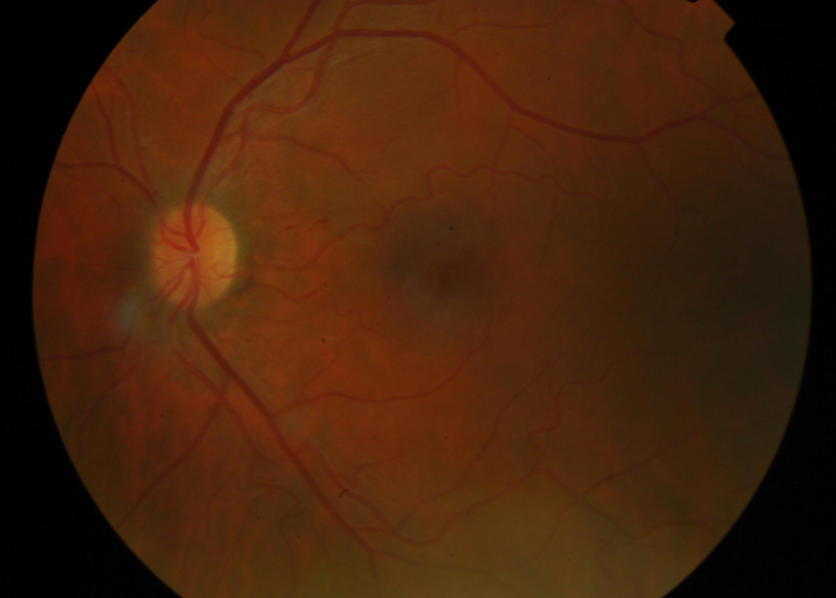
Color fundus photograph of the left eye, at one-month follow up, showing regression of almost all retinal lesions

We report a 39-year-old caucasian man who has lived in Angola for 4 years. He presented to a hospital in California, during a trip, with a flu-like syndrome, jaundice and altered state of consciousness. On hospital admission, he had hemolytic anemia, thrombocytopenia, acidemia and acute renal failure. After the diagnosis of *Plasmodium falciparum* malaria, the patient initiated therapy with quinine and doxycycline, although developing severe encephalopathy (coma). After therapy with artesunate, he had hematologic and neurological improvement, and was transferred to Portugal. On initial eye examination at our hospital, he had 20/20 visual acuity in the right eye and a central scotoma in the left eye. Right eye indirect ophthalmoscopy revealed round deep layer retinal hemorrhage, with one disc diameter of radius, in the superior temporal arcade (Figure 1 (Fig. 1)). Left eye indirect ophthalmoscopy showed a foveal hemorrhage, several superficial hemorrhages in the interpapillomacular bundle (Figure 2 (Fig. 2)) and a deep white-centered peripheral hemorrhagic lesion (Figure 3 (Fig. 3)). Fluorescein angiography was not performed given the deterioration of the renal function warranting hemodialysis. At one-month follow-up, the patient had 20/20 visual acuity in both eyes and on examination almost all lesions shown on retinography had regressed (Figure 4 (Fig. 4) and Figure 5 (Fig. 5)).

## Discussion

Changes defined as malarial retinopathy were first described in 1993, by Lewallen et al. in Malawian children [3]. Although mostly described in children, this ophthalmologic manifestation also occurs in adults. It is currently the most sensitive and specific clinical sign of cerebral erythrocyte sequestration, the cardinal histopathologic feature of cerebral malaria [2]. The main importance of the identification of its retinal features lies in the fact that the systemic clinical manifestations are not specific for this disease and also, sometimes, the laboratory diagnosis may not be available, especially in malaria endemic areas [2].

Despite being more frequent in cases of encephalopathy, retinopathy can rarely occur in cases of *Plasmodium falciparum *and* Plasmodium vivax* malaria without brain involvement [1]. On the other hand, the absence of this finding in cases of encephalopathy with altered state of consciousness must encourage the investigation of other causes of the symptoms, such as trypanosomiasis, herpes simplex encephalitis, Reye’s syndrome and poisoning, in order to provide appropriate early treatment [2].

Malarial retinopathy is characterized by three main fundoscopic manifestations:

1. Retinal whitening; this occurs more commonly in the macular and perifoveal area, and corresponds to areas of retinal hypoperfusion caused by sequestered parasitized erythrocytes in the retinal microvasculature, leading to loss of transparency of the hypoxic tissue. The severity of retinal discoloration has a direct correlation with the amount of serum lactate measured on admission [1], [2].
2. Vascular changes; consists of vascular discoloration, primarily at the level of the capillaries, more frequent in peripheral areas of the retina [2].
3. Hemorrhages; the most common retinal hemorrhages of malarial retinopathy show a white central area that corresponds to the presence of fibrinoid matter [2]. The amount of hemorrhages is related not only to the severity of underlying disease, but also to the patient’s degree of anemia and number of brain hemorrhages [1], [4].

Papilledema is present in some cases, indicating a severe underlying disease; nevertheless, it is not a specific disease characteristic [2]. Also, neurological sequelae of malarial retinopathy occur in about 12% of children, with subsequent resolution of the majority of the cases, and in 1% of adults [5]. There are no systematic reviews about the effect of retinal changes in visual acuity during the acute phase of the disease, although Beare et al. have concluded that there is no correlation between the severity of retinopathy and visual acuity at day 30 after diagnosis in children [6], [7].

In this case report, superficial hemorrhagic lesions in the interpapillomacular bundle confirm vascular changes in this area, as well as the mentioned deep hemorrhages. The absence of discoloration of retinal vessels is in agreement with Maude et al., who did not found this change in any of the studied adults [1]. The patient showed full recovery of visual acuity associated with almost complete regression of retinal hemorrhagic lesions. Probably the identification of the ocular features in this patient would have provided an earlier specific therapy and the avoidance of the severe encephalopathy development, although one cannot state it certainly. Thus, this specific case highlights that the identification of the ophthalmological manifestations in patients with similar systemic clinical presentation may provide an earlier diagnosis and avoid severe complications of malaria. 

## Conclusion

Malarial retinopathy is a diagnosis factor of severe *P. falciparum* infection, usually associated with brain involvement. In addition, it constitutes a prognosis indicator, since retinal changes correlate with the severity of the underlying condition, duration of altered state of consciousness and mortality. 

In view of being an easily recognizable manifestation and a possible sign of malarial encephalopathy, the knowledge of the ophthalmological characteristics associated with this disease and its identification, is crucial itself as it leads to the early diagnosis of cerebral malaria, given that neither the systemic manifestations are specific to malaria nor the laboratory diagnosis investigation is always available, especially in endemic areas [2]. Thus, when the diagnosis is suspected it conducts to the initiation of specific systemic treatment for this disease, avoiding the possible complications of this condition with a delayed treatment, such as neurologic sequelae and associated mortality. Also, as a prognosis factor, it will allow targeted therapy, according to the severity of the disease. 

Therefore, this case emphasizes that in cases with similar clinical presentations, and in the presence of encephalopathy, fundus examination should be routinely performed, especially in patients from endemic areas, either children or adults.

## Notes

### Informed consent

The patient described in the case report has given his informed consent for the case report to be published.

### Competing interests

The authors declare that they have no competing interests.
